# Older adults’ adoption of shared service platforms outside long-term care insurance in Japan based on technology acceptance model

**DOI:** 10.3389/fpubh.2026.1847810

**Published:** 2026-07-23

**Authors:** Chao Li, Chunji Zheng, Ziheng Lv

**Affiliations:** 1 School of Business, Guilin University of Technology, Guilin, China; 2 School of Social Welfare, Tokyo University of Social Welfare, Tokyo, Japan

**Keywords:** behavioral intention, long-term care insurance, LTCI, older adults, sharing economy, TAM, technology acceptance model

## Abstract

The services that are not covered by the long-term care insurance play a critical role in supplementing the public care system in the aging society of Japan. Their combination with technologies of the shared economy is not only an extension of the shared economy into the specialized service market, but it is also a natural demand of the market in the long-term care service market that is the necessity to innovate and develop. This study constructed a technology acceptance extension model, using the Tokyo area as the survey scope. 486 valid samples were collected through a questionnaire survey, and partial least squares structural equation modeling was used to conduct hypothesis testing and empirically analyze the acceptance behavior of the older adult towards shared service platforms outside of long-term care insurance. The main findings are: (1) Scene characteristics may have a reshaping effect on the local behavior of the older adult in technology acceptance. (2) The single cognitive load of new technology in old scenes and the dual cognitive load of new technology in new scenes may be key to whether the older adult technology acceptance behavior undergoes local reshaping. (3) The “first principle” of perceived ease of use in the cognitive logic of the older adult group is emphasized. (4) The “external social construction” of perceived usefulness and the “endogenous individual defense” of perceived risk may be the unique cognitive logic of acceptance behavior in such scenarios.

## 1 Introduction

The sharing economy has been permeating into the conventional industry frames with its unique resource distribution patterns and value generation processes due to the ever-changing nature of digital technologies and radical shifts in socioeconomic frameworks (1). It has creeped into high-barrier specialty domains such as healthcare (1–5). Technological change is driving social transformation, profoundly impacting the healthcare sector, particularly long-term care services. Long-term care services are closely intertwined with the daily lives of older adult in Japan, and how to guide them to proactively adapt to new industry changes and help them alleviate digital inequality is a topic of close interest to scholars (6, 7).

Japan’s previous single-mode older adult care system, which relied primarily on long-term care insurance (LTCI) services, has been broken. The rapid development of services outside of LTCI has become an important supplement for seniors to age in place and achieve successful aging. Since the LTCI system was officially introduced in 2000, the Japanese government saw the need to invoke systematic policy changes as the number of the older adult grows in population thus putting fiscal strains on their budgets (8–11). These comprised modifying the cost-sharing tool of LTCI via modifying enrollee ratios of the copayment, the range of service coverage and the duration and frequency of the service (9, 12, 13). Additionally, the 2016 “Flexibilization of Mixed Care Services” policy (15) was launched, which diversifies the supply of long-term care services by breaking the monopoly of supplying the public services. Through these efforts, older adults have gradually become aware that with increasing age and increasing care demands, the shortcomings of traditional LTCI services in terms of coverage breadth and service depth will become more visible. Relying solely on existing services is insufficient not only to guarantee quality of life but also to achieve successful aging (13). That is why non-LTCI services have become an indispensable complement to home-based older adult care (13), and the demand in the market has a massive impact on the development of the industry. The services market outside of LTCI has shown a healthy growth and this has exceeded 77 trillion yen (around $514.1 billion) by 2050 according to the Action Plan 2023 of a New Healthy Society by the Ministry of Economy, Trade and Industry (16).

Nevertheless, the traditional older adult care service industry is often constrained from rapid development by factors such as the lack of professional caregivers, slow response of the provided services, and the lack of adjustment to individual needs (17–19). The sharing economy technology will lead the transformation of LTCI services from traditional services to digital services, injecting new momentum into the industry’s development. It successfully breaks through the geographical barriers and information barriers using its main strengths of efficient resource integration and flexibilities in the provision of services (4, 5) and opens new avenues to the digital transformation of other services not delivered by LTCI. Such models as shared service models such as Ichirou company^1^ create internet technology platforms to enable accurate delivery of fragmented care service providers to the demand side of the older adult. This creatively incorporates the fragmentation and underutilization of care resources, which is important in enhancing the efficiency of resource use and provides a feasible solution to the varied and customized care requirements of older adults. Japanese seniors may be able to age successfully by accessing a shared service platform outside of long-term care insurance, represented by this company.

While shared service platforms outside of LTCI offer numerous advantages in optimizing the allocation of older adult care resources, the widespread digital inequality among older adults may hinder their adoption of these platforms (6, 20, 21). Digital inequality has long surpassed the primary level of access inequality, evolving into a higher level of skills and usage inequality. Even with identical devices and internet access, varying levels of digital literacy can lead to differences in acceptance of the shared service platform. This inequality in outcomes, stemming from skills and usage inequality (6, 20, 22), may be the underlying cause of the significantly lower-than-expected actual adoption rate of these shared service platforms^2^. Therefore, how to overcome the challenge of Japanese older adult acceptance of shared service platforms outside of LTCI under the new circumstances, and help them adapt to the changing trends of long-term care services and achieve successful aging, has become an urgent and important research topic.

In the healthcare field, current academic research on these issues still has many gaps. First, although online sharing platforms are not new technologies, their deep integration with LTCI-exclusive services has created innovative application scenarios for the older adult. Existing research on LTCI-exclusive services largely focuses on traditional areas such as industry development, service standards, and service content (23–26), with less attention paid to the integration and innovation of this service with sharing economy technologies, creating a research gap. Second, while some scholars have noted the value of the sharing economy in promoting long-term care services, related studies mostly employ qualitative analysis, lacking systematic quantitative empirical testing, and failing to delve into the technology acceptance behavior and underlying cognitive logic of the older adult (3), which also constitutes a research gap. Finally, a review of relevant literature reveals that in research on technology acceptance among the older adult in the healthcare field, the fit of the Technology Acceptance Model (TAM) may be correlated with the specific research scenario. Specifically, the behavioral intentions of older users may be unaffected by perceived ease of use (27–29) and also do not affect perceived usefulness (30, 31), while perceived ease of use does not necessarily affect perceived usefulness (28). Furthermore, the empirical results of the extended TAM model, which incorporates variables such as social influence, self-efficacy, individual innovativeness, and perceived risk, do not necessarily meet expectations (32–36). This reflects that the acceptance behavioral intentions of older adults towards various new technologies is not static, and some of their behavioral patterns may be influenced by specific scenarios. However, existing research often fails to consider the differences in service acceptance patterns across different scenarios and neglects the shaping effect of scenario characteristics on behavioral intentions. This is the key gap that this study aims to fill.

Based on the aforementioned research gaps, this study will focus on the acceptance behavioral intentions of the Japanese older adult towards shared service platforms outside of LTCI in a new scenario, and proposes the following research questions (RQs): RQ1: In the new scenario of shared services outside of LTCI, what are the characteristics of the technology acceptance behavioral intentions of the Japanese older adult? What are the core influencing factors driving their intention to use the platform? RQ2: Through what internal mechanisms do the above core factors influence their technology adoption decisions? What is the cognitive logic behind their technology acceptance behavioral intentionsr? RQ3: How can we systematically extract and explain the complex cognitive logic of technology acceptance among the older adult to advance related theoretical development and provide theoretical reference for improving digital inequality among the older adult?

To address the aforementioned research questions and fill existing research gaps, this study follows an overall approach of “empirical testing - cognitive perspective - theoretical dialogue.” This paper uses shared services outside of LTCI as the research scenario, constructing an analytical framework for technology acceptance among the older adult based on the TAM. Through empirical testing, it identifies the core factors influencing the acceptance of this shared service platform by the older adult in Japan. Building upon this foundation, it delves into the underlying cognitive logic of older adult users’ technology acceptance behavior in this specific scenario and discusses it, providing theoretical support and reference for alleviating digital inequality among the older adult in the shared service industry outside of LTCI from a behavioral perspective.

Based on the aforementioned research approach, this study will review its contributions from both theoretical and practical perspectives. Theoretically, this study keenly focuses on the subtle differences behind the apparent similarities in technology adoption behavioral intentions among the older adult, aiming to address the challenge of localized behavioral variations in technology acceptance across different scenarios. Differences in service scenario cognition may be the core reason for the localized differentiation in technology acceptance behavior among the older adult; that is, scenario characteristics may have a localized reshaping effect on their technology acceptance patterns. Simultaneously, this study dialectically integrates the contextual logic of contingency theory and the general explanatory power of the TAM. Combined with empirical results, it verifies their compatibility and rationality of integration within the scenario of age-friendly emerging services, thereby refining the contextual boundary interpretation of existing theories. In practice, this study, combining theoretical conclusions, provides systematic management insights for the operation of age-friendly technology platforms. The study points out that managers need to consider industry and service attributes, fully recognizing that localized technology acceptance behaviors of older adult users may be reshaped by scenario cognition. Therefore, it is necessary to implement dual cognitive management, taking into account both technology cognition and service cognition, and dynamically optimize operational strategies. In promotion practice, enterprises should prioritize perceived ease of use, relying on the concept of “integrated communication” to simultaneously simplify the understanding threshold of service scenarios and the difficulty of platform operation, alleviating the dual cognitive anxiety of the older adult. At the same time, enterprises should adopt a precise promotion model, leveraging social word-of-mouth for external guidance, cultivating and activating users’ intrinsic motivation through digital capabilities, and building a risk control system to eliminate usage concerns. A systematic operational strategy can reduce promotion costs, break down implementation barriers, and promote the long-term popularization of older adult service platforms.

The remaining sections of this article are organized as follows: Section 2 provides the theoretical background, and Section 3 elucidates the conceptual model and associated research hypotheses. Sections 4 and 5 are dedicated to the methodology and presentation of results, respectively. Finally, Section 6 contains the discussion, outlines the implications, and addresses the study’s limitations, leading to the conclusions presented in Section 7.

## 2 Theoretical background

### 2.1 Shared service platforms outside LTCI

Being a platform model that is based on information technology, the sharing economy is at its core about shared underutilized assets or services among people (37). It has gained a lot of and long-lasting scholarly interest, being pushed forward by digital technologies (38). The sharing economy is also accurate in matching the suppliers of goods or services with the demanders through web-based channels, and the idle or poorly used resources are used within the society. This establishes effective interaction channels between the supply and demand to facilitate a sequence of essential activities of quick matching, convenient search, even flow of communication and secure transactions (39). Currently, the scope of the sharing economy is continuously expanding, having fully permeated various industries beyond the initial domains of accommodation and transportation (2), significantly influencing even the long-term care service sector (1, 3).

From the perspective of older adults as service users, long-term care services can be categorized into services inside LTCI and services outside LTCI. The latter plays a vital complementary role in the overall long-term care system (13). These services fall outside the scope defined by the LTCI system, and the service costs must be fully borne by the service users themselves (15, 25). In terms of scope and content, services outside LTCI primarily include (14, 25): (1) Daily living support services include housekeeping, outing companionship, pet care, gardening and administrative procedure assistance, with customized solutions based on “home situation diagnostics”; (2) Health prevention services include literacy training, cognitive games, and exercise classes designed to maintain good physical function and delay aging symptoms; (3) Social engagement services provide emotional companionship, intergenerational exchange, and customized travel arrangements, aimed at helping older adults expand their social networks and reduce loneliness; (4) Safety monitoring services provide real-time surveillance and regular visits, linked with older adults’ health data, to acutely detect abnormal situations and promptly trigger emergency response mechanisms, ensuring life safety.

The inorganic connection between services not within LTCI and the sharing economy is not only a natural tendency in the comprehensive evolution of the sharing economy, but also a necessity in the innovation and growth of the market of long-term care services (3). Basically, shared services not included within LTCI lie in the specialized skill-sharing, which is highly intensive by either sharing the intangible resources like knowledge, skills, and time (40). These services in this research are clearly stated as: a model of service that involves the use of internet to perfectly match and distribute idle intangible resources- such as skills, abilities, and time- with older adults that require long term care.

However, the online technological platforms and applications involved in these shared services may be challenging for older adults who are accustomed to offline services and less proficient with new technologies (41). More likely, inequalities in skills and usage, and consequently, inequalities in outcomes, hinder the development of out-of-insurance long-term care services. Therefore, in this emerging context, this study delves into the core factors influencing older adults’ adoption of such technologies and their underlying cognitive logic, offering a theoretical explanation from an older adult behavior perspective and guiding effective corporate management to alleviate digital inequality among older adults.

### 2.2 Technology acceptance model (TAM)

In the context of research on shared services outside of LTCI, the primary task in accurately identifying the core factors and behavioral characteristics of older adult technology acceptance behavior is to select an appropriate technology acceptance model. In the healthcare field, the analysis of older adult technology acceptance behavior often relies on classic frameworks such as the Technology Acceptance Model (TAM) and the Technology Use and Adoption Theory (UTATU) (30, 42–45). Since this paper mainly explores the fundamental variables that affect technology acceptance behavior and the mechanism of the older population. Crucially, it does not involve differential impact analysis of demographic constructs such as age, gender, or previous technical experiences (44). Therefore, the TAM was chosen as the theoretical cornerstone for constructing the empirical research framework.

The TAM, conceptualized by Davis in 1986 and rooted in the theory of reasoned action(TRA), offers a structured theoretical lens for anticipating and comprehending how users embrace information technology (46). Perceived ease of use and perceived usefulness are two important factors in the TAM foundation for determining systems understanding. Perceived ease of use indicates how easy a system is to use, while perceived effectiveness measures how much a customer believes a particular technology will improve their work performance. These two elements jointly shape a user’s intention to engage with novel technology (46). Also, there is a heavy positive correlation between perceived ease of use and perceived usefulness since more individuals are inclined to perceive usefulness of easy-to-use and easy-to-learn technology (47). The user attitude was also originally a relationship between the perceived ease of use, perceived usefulness and behavioral intention but this factor was deliberately excluded in future studies (46). Therefore, this study will select perceived ease of use, perceived usefulness, and behavioral intention as core variables to conduct empirical research.

In the healthcare field, TAM-based behavioral studies on older adults are quite extensive (48, 49). However, a literature review of research scenarios such as smart health devices (31, 50, 51), mHealth applications (27, 52, 53), and eHealth services (28, 30) revealed different results when exploring older adults’ acceptance of new technologies. Older adults’ behavioral intention may not be significantly positively influenced by perceived usefulness (30, 31), nor by perceived ease of use (27, 28). Additionally, perceived ease of use does not always positively influence perceived usefulness (28). These inconsistent findings suggest that older adults may exhibit different behavioral outcomes and acceptance patterns when faced with different, specific new technology services. Therefore, this study needs to empirically test the applicability and stability of TAM in a new context.

Since a single TAM model is insufficient to fully explain the complex technology acceptance behavior of older adults, it is necessary to simultaneously consider the significant roles of environmental factors and individual characteristics in influencing the technology acceptance process (54, 55). This study agrees with the approach of incorporating key variables such as social influence, self-efficacy, personal innovativeness, and perceived risk into the TAM to jointly construct the research model (29, 35, 43, 45, 56–60). This research model can more comprehensively analyze the acceptance behavior of older adults towards shared service platforms outside of LTCI and its underlying mechanisms, and attempts to explain the deep cognitive logic behind the behavioral manifestations from a more macro perspective, which can provide strong theoretical support for alleviating digital inequality among older adults.

## 3 Conceptual model and research hypotheses

**Figure 1:**
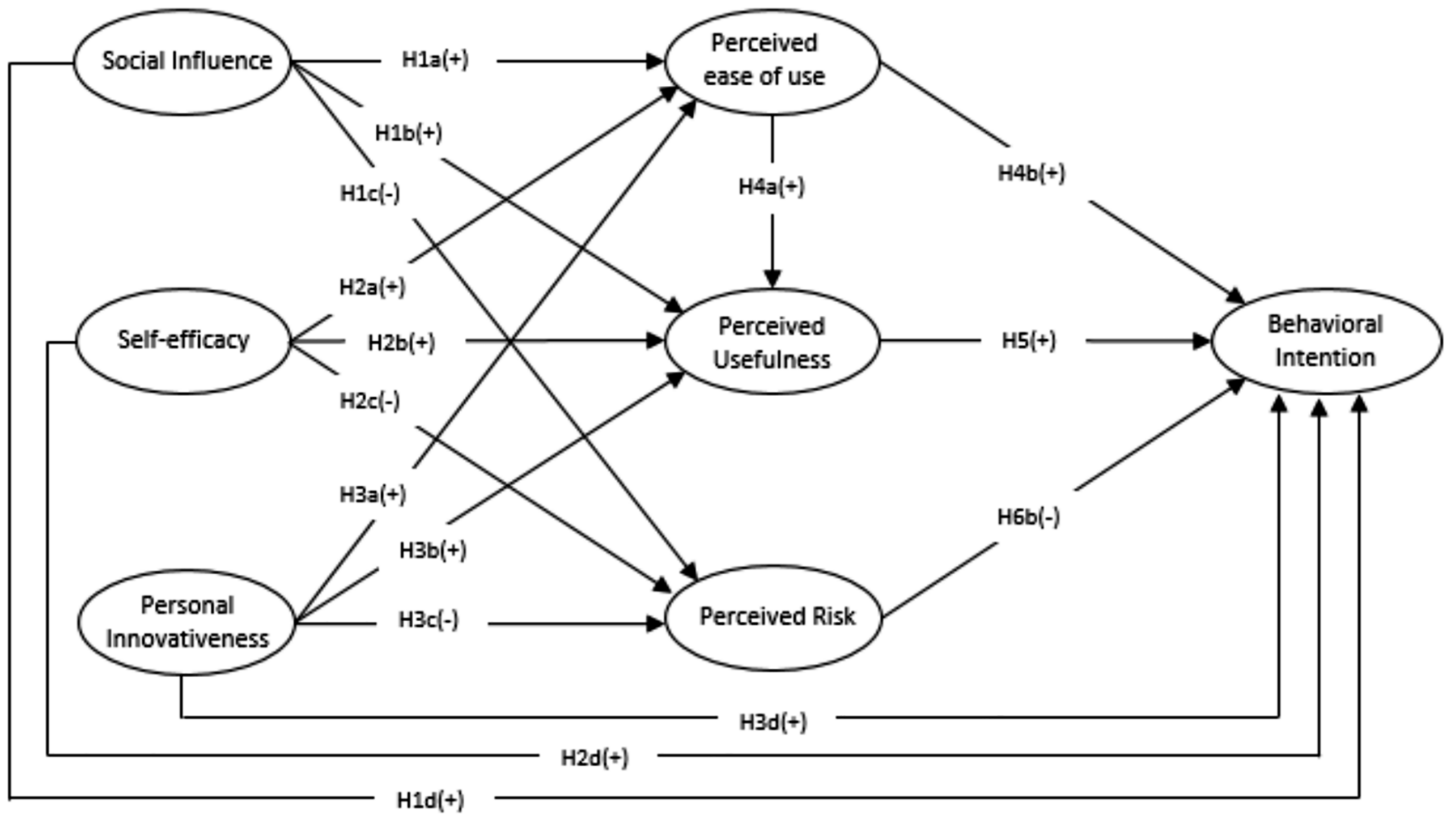
Research model.

As mentioned earlier, this study will construct a research model using seven variables: perceived ease of use, perceived usefulness, behavioral intention, social impact, self-efficacy, personal innovativeness, and perceived risk (as shown in Figure 1) to empirically demonstrate the psychology and behavioral intentions of older adults when accepting shared services platforms outside of LTCI. The model suggests a cumulative amount of 16 hypothesis of the research. The social influence and self-efficacy, and personal innovativeness variables can be considered both as environmental and individual factors. Older adults are characterized by psychological perception states that are captured using perceived ease of use, perceived usefulness, and perceived risk. The outcome variable is behavioral intention which is the attitude of older adults toward new technologies. This study uses this well thought-through research model to analyze the role of such factors as social influence, self-efficacy, and personal innovativeness in the ultimate formation of the behavioral intention in the older adult through the prism of their perceptions of ease of use, usefulness, and risk.

### 3.1 Social influence

The attitudes and behaviors of other people often affect the choices of people which is a phenomenon referred to as social influence and a major contributor of behavioral intention (61). The influence of social factors on the uptake of the new technological services offered by the healthcare industry is also considered to be a significant factor among the older adults (45, 62). The implementation of new technological services by family and friends can reduce the expectations of the older adults regarding the difficulty of the use of the services and enhance the user experience and perceived ease of use. Furthermore, the recognition of the service can make older people perceive the usefulness of the service more (63–65). Moreover, the perceived risk of using the service will be lower when a user is positively affected by others, including his or her friends and relatives (66). Such reliable relationships significantly lessen user uncertainty when using new services and reduce perceived risk (67, 68). Therefore, this study hypothesizes:

*H1a*: Social influence has a significant positive impact on older adults' perceived ease of use of shared service platforms outside LTCI.

*H1b*: Social influence has a significant positive impact on older adults' perceived usefulness of shared service platforms outside LTCI.

*H1c*: Social influence has a significant negative impact on older adults' perceived risk of shared service platforms outside LTCI.

*H1d*: Social influence has a significant positive impact on older adults' behavioral intention to adopt shared service platforms outside LTCI.

### 3.2 Self-efficacy

Self-efficacy refers to a belief in the ability of an individual to perform certain tasks or to achieve certain goals (69). Self-efficacy is theorized within the context of this study by the belief that older adults have towards successfully using the internet and application software to access these services. Current literature proves that self-efficacy positively and significantly influences perceived usefulness, perceived ease of use, and behavioral intention of the older adult, encouraging them to accept and use new services on technology (32–34, 70). In addition, high self-efficacy individuals tend to make underestimates and solutions and generally believe that they are capable of coping with problems; low self-efficacy individuals, on the contrary, make overestimates and adopt conservative attitudes, which affect adoption decisions (68, 71, 72). Therefore, this study hypothesizes:

*H2a*: Older adults' self-efficacy has a significant positive impact on the perceived ease of use of shared service platforms outside LTCI.

*H2b*: Older adults' self-efficacy has a significant positive impact on the perceived usefulness of shared service platforms outside LTCI.

*H2c*: Older adults' self-efficacy has a significant negative impact on the perceived risk of shared service platforms outside LTCI.

*H2d*: Older adults' self-efficacy has a significant positive impact on the behavioral intention to adopt shared service platforms outside LTCI.

### 3.3 Personal innovativeness

One of the concepts of consumer behavior that is significant is personal innovativeness (73), which is a natural urge of individuals to adopt and experiment on new technologies (74). Those revealed to display greater degrees of this characteristic tend to be much more willing to adopt and embrace new technologies and services (36). Highly innovative older adults in the healthcare field are likely to actively seek health information on the Internet (35). They do not mind wearing their health observation gadgets (56), which means that personal innovativeness is a strong selling point when it comes to technology acceptance intention. Furthermore, a negative correlation between personal innovativeness and perceived risk has been observed in various fields, including service robots (75), 3D printing (76), and shared bicycles (77). This suggests that very impressive individuals demonstrate a large degree of openness and acceptance toward fresh things, are fearless in trying new technologies and methods, and have a high tolerance for uncertainty and a risk-taking attitude. Therefore, this study hypothesizes:

*H3a*: Older adults' personal innovativeness has a significant positive impact on the perceived ease of use of shared service platforms outside LTCI.

*H3b*: Older adults' personal innovativeness has a significant positive impact on the perceived usefulness of shared service platforms outside LTCI.

*H3c*: Older adults' personal innovativeness has a significant negative impact on the perceived risk of shared service platforms outside LTCI.

*H3d*: Older adults' personal innovativeness has a significant positive impact on the behavioral intention to adopt shared service platforms outside LTCI.

### 3.4 Technology acceptance model (TAM)

TAM is a theoretical framework for understanding individual technology usage behavior in particular circumstances (78). Users are more likely to adopt and use a technology or service when it is deemed useful and simple (78). The world’s aging necessitates more older adults using innovative technological services, making a study on older adult actions based on technology acceptance particularly important. Reports show that TAM is commonly applied in the healthcare services website (30, 31, 48, 53) and possesses explanatory power for older individuals ‘systems understanding. Therefore, this study hypothesizes:

*H4a*: Older adults' perceived ease of use has a significant positive impact on the perceived usefulness of shared service platforms outside LTCI.

*H4b*: Older adults' perceived ease of use has a significant positive impact on the behavioral intention to adopt shared service platforms outside LTCI.

*H5*: Older adults' perceived usefulness has a significant positive impact on the behavioral intention to adopt shared service platforms outside LTCI.

### 3.5 Perceived risk

Perceived risk is an individual’s subjective judgment of potential unfavorable consequences or losses before making a decision or taking a specific action (79). This risk is not the objective existence, but rather an individual’s perception and assessment of potential risks based on their own experience, knowledge, preferences, and understanding of the situation (80). Older adults tend to be more cautious when facing new technological services, relying on past experiences to evaluate things. Faced with numerous uncertainties associated with new technologies, they may find it difficult to master and cope with, leading to resistance. Ultimately, to avoid potential losses and troubles, their purchase intention decreases (60, 65, 81, 82). Therefore, this study hypothesizes:

*H6*: Older adults’ perceived risk has a significant negative impact on the behavioral intention to adopt shared service platforms outside LTCI.

## 4 Methodology

This will be followed by an empirical study that will be done systematically and will involve major steps in the entire process of the investigation and these will include development of questionnaire, data collection, sample description, and data analysis procedure in order to thoroughly investigate the relationships among the latent variables in the hypothesized research model. In addition, this study adopts a cross-sectional survey design.

### 4.1 Questionnaire development

This study questionnaire is mostly divided into two parts. The former has a scale of 27 measurement items that depict the seven latent variables of the research model, namely: social influence, self-efficacy, personal innovativeness, perceived ease of use, perceived usefulness, perceived risk, and behavioral intention. The second section gathers demographic data regarding the respondents, namely; gender, age, level of education, and marital status.

To fit the present research setting, scales in previous valid and tested researches were modified in this study. Table 1 presents all latent variables, measurement items and respective sources of writing. The measurement items were analyzed in a five-point Likert scale between 1 (Strongly Disagree) and 5 (Strongly Agree). In this paper, the back-translation technique was employed in producing a questionnaire that was formed within the Japanese linguistic framework (83) since the original measures were prepared in English.

**Table 1:** Measurement instruments.

Construct	Items	Measurement	Source
Social influence	SI 1	People who are important to me think that I should use services outside LTCI through online sharing platforms.	(61, 64, 65)
	SI 2	People around me think it is a good idea for me to use services outside LTCI through online sharing platforms.	
	SI 3	People who use services outside LTCI through online sharing platforms look more capable than those who do not.	
	SI 4	The use of services outside LTCI through online sharing platforms is a trend. I want to keep up with the pace of the times.	
Self-efficacy	SE 1	I think I can learn to use services outside LTCI through online sharing platforms if I put in the effort.	(32, 33, 98)
	SE 2	I think I can complete the operation of online sharing services outside LTCI by reading the manual.	
	SE 3	I think I have the necessary skills to use services outside LTCI through online sharing platforms.	
	SE 4	I think I know how to use services outside LTCI through online sharing platforms.	
Personal innovativeness	PI 1	I think it is fun to try new information technology.	(35, 56, 74)
	PI 2	I am willing to try new information technology.	
	PI 3	If I heard about a new information technology, I would look for ways to experiment with it.	
	PI 4	Among my peers, I am usually the first to try out new information technologies.	
Perceived ease of use	PEU 1	I find it easy to get services outside LTCI through online sharing platforms to do what I want to do.	(61, 78, 99)
	PEU 2	My interaction with services outside LTCI through online sharing platforms is clear and understandable.	
	PEU 3	I find services outside LTCI through online sharing platforms to be flexible to interact.	
	PEU 4	I find services outside LTCI through online sharing platforms to be easy to use.	
Perceived usefulness	PU 1	I think that using services outside LTCI through online sharing platforms makes life more convenient.	(61, 78, 99)
	PU 2	I think that using services outside LTCI through online sharing platforms can improve efficiency.	
	PU 3	I think that using services outside LTCI through online sharing platforms to be useful in life.	
	PU 4	I think that using services outside LTCI through online sharing platforms improves my quality of life.	
Perceived risk	PR 1	I’m worried that my privacy will be leaked when I use services outside LTCI through online sharing platforms.	(60, 64, 82)
	PR 2	I’m worried that my health data will be misused when I use services outside LTCI through online sharing platforms.	
	PR 3	I’m worried that I will suffer property losses when I use services outside LTCI through online sharing platforms.	
Behavioral intention	BI 1	I intend to use services outside LTCI through online sharing platforms in the future.	(61, 82, 99)
	BI 2	When I have health problems, I am willing to use services outside LTCI through online sharing platforms.	
	BI 3	I think that services outside LTCI through online sharing platforms are essential for me.	
	BI 4	I would like to recommend services outside LTCI through online sharing platforms to others.	

### 4.2 Data collection and sample description

As a core city for innovation and development in Japan, choosing the Tokyo area as the sampling region is reasonable, as it aligns with the research theme of acceptance of emerging technologies. Furthermore, the Tokyo area has a large and densely populated older adult with a high level of awareness regarding services outside of LTCI, providing a representative sample and reliable research data for this study.

This survey employed stratified and convenience sampling methods. Given Tokyo’s status as a popular tourist city, the stratified sampling phase deliberately avoided popular tourist attractions and areas with high tourist density, prioritizing areas with concentrated local residents and a strong sense of community. After determining the sampling area, intercept surveys were conducted at shopping malls, train stations, parks, and senior welfare centers within the jurisdiction, with all survey activities requiring prior consent from site management. Before conducting the questionnaire survey, each respondent was clearly informed of the academic purpose of the study, the specific content of the survey, the principle of complete anonymization of data, and the privacy protection commitment. The survey was only conducted after obtaining their voluntary consent. Considering that a very small number of older adult (no more than 4% of the total questionnaires) might have reading and writing difficulties, data was collected through a process where investigators read the questions aloud, respondents answered orally, and investigators filled out the questionnaires on their behalf. During the questionnaire collection process, investigators maintained a neutral attitude and did not make any subjective or leading statements to ensure the authenticity and validity of the survey data. Two certified investigators were included on the field team to provide a systematic collection of data. Data collection was conducted from January to March 2025, collecting 593 questionnaires from older adult aged 50 and above^3^. After removing invalid questionnaires with patterned responses, incomplete responses, or ineligible participants, 486 valid samples were obtained. In addition, considering that people aged 79 and above may experience cognitive decline and have lower sensitivity to information technology and emerging services, making them prone to answering biases and unable to provide effective feedback, this age group was excluded from the survey^4^.

This study also assumed a sufficient level of statistical power, as it was not based on the 10- rule” (or 5- rule) (84); however, Cohen method was used to calculate a minimum required sample size of 207 (seven predictors and the effect size of 0.07) (85, 86). This outcome is far much less than the respective effective sample size produced thus guaranteeing accuracy and credibility to successive statistical analysis.

The demographic profile of the sample is provided in a detailed way in Table 2. The results of the analysis are as follows: males were 56.2 percent of the participants, and the female population was 43.8 percent. The age structure indicated a 43.0% of 50–59 years, 37.9% of the 60–69 years and 19.1% of 70–79 years. The level of education indicated that 64.8 percent had high school education and below, 21.0 percent had a junior college education, 10.5 percent had a bachelor degree, and 3.7 percent had a postgraduate degree. Lastly, on the marital status, 75.1 percent of the respondents were married with only 24.9 percent being single.

**Table 2:** Demographic information (*n* = 486).

Characteristics	Categories	Frequency	Percentage
Gender	Male	273	56.2%
	Female	213	43.8%
Age	50–59	209	43.0%
	60–69	184	37.9%
	70–79	93	19.1%
	Over 79	0	0%
Education level	high school and below	315	64.8%
	junior college degree	102	21.0%
	Undergraduate degree	51	10.5%
	Postgraduate degree	18	3.7%
Marital status	Married	365	75.1%
	Single	121	24.9%

### 4.3 Data analysis procedure

Structural equation modeling (SEM) is one of the widely used approach to empirical research, and it includes covariance-based SEM (CB-SEM) and partial least squares SEM (PLS-SEM) (87). Scholars Hair et al. (87) argue that there are distinguishable practical differences between the two modelling approaches in hypothesis testing. The core objective of CB-SEM is to validate or refute established theories; it verifies model validity by examining the goodness-of-fit between the theoretical model and the covariance matrix derived from sample data. In contrast, PLS-SEM estimates relationships among latent variables based on the variances of all observed indicators, prioritises the prediction of dependent variables, and is characterised as a causal-predictive analytical method. This method presents prominent advantages for predictive modelling and exploratory analysis of complex extended frameworks. Given that the empirical objective of the present study is to uncover causal linkages across constructs and evaluate their predictive efficacy, PLS-SEM is selected, and SmartPLS 4.1 is adopted for statistical analysis.

The two-stage analysis paradigm of SEM (88) is strictly followed in this study because the analysis involves first having an in-depth analysis of the measurement model and then analysis of the structural model to confirm that the model is rigorous and scientifically valid. The study will critically discuss the collinearity problems, the elements loading, and the levels of values of every variable at the stage of the measurement model investigation and will also assess their stability, convergent validity and discriminant validity to confirm the strength and accuracy of the measurement model. The testing of the structural model will then look at the various assumptions used in the study design in isolation. It will be measured using three key variables (coefficient of determination (R^2^), effect size (f^2^), and cross-validated redundancy (Q^2^)) (89).

## 5 Results

### 5.1 Common method bias

As the data were gathered using questionnaires, the common method bias (CMB) was a possible issue (90). A preliminary evaluation of CMB was done before the measurement model analysis using the single-factor test of Harman. The findings revealed that only one factor was found to account 48.6% of the overall variance which is less than 50 percent (91).

Nevertheless, due to the fact that Harman single factor test is not very sensitive in identifying CMB (91), the more rigorous single-label technique (92) was utilized in this study as well. Comparison of models of presence and absence of the marker variable revealed that the differences between the standardized weights of the regression of all the measurement items are less than 0.2. Also, the variations in correlation coefficients between all latent variables were also minimal and four-tailed insignificant (93). All these analyses uphold the fact that CMB is not a significant issue in the data gathered. This indicates that the pre-test controls in this study were scientific and standardized, the questionnaire measurement items were clearly described, which made it easy for respondents to accurately understand the questions and effectively ensured the reliability of the subsequent model testing.

### 5.2 Evaluation of the measurement model

The measurement model was evaluated systematically with the data satisfying all the preset criteria. The Table 3 shows that the variance inflation factors (VIFs) were all less than 5, which proves that there was no multicollinearity in the model (89). On top of this, all the factors loading were more than 0.708, and the values of Cronbach alpha, composite reliability (CR), and average variance extracted (AVE) were greater than their values (89). These statistics all testify to the reliability and convergent validity of our information. This indicates that the respondents’ responses were consistent and stable, and that each measurement item could effectively correspond to the latent variables, which provides the possibility for constructing the influencing factors needed for a theoretical model. In order to achieve discriminant validity, Fornell-Larcker criterion and heterotrait-monotrait (HTMT) ratio were used in this research. As it can be seen in Table 4, the data below the diagonal are all smaller than the square root of AVE on the diagonal (94), and the data above the diagonal are all below 0.85 (95). These reliable results clearly indicate that there is high discriminant validity of data utilized in this study. The clear conceptual boundaries and independence of the variables provide a reasonable basis for the causal path relationships between them in the subsequent theoretical model.

**Table 3:** Measurement model.

Construct	Items	Loadings	VIF	α	CR(rho_a)	CR(rho_c)	AVE
Social influence	SI 1	0.924	4.017	0.930	0.931	0.950	0.827
	SI 2	0.907	3.475				
	SI 3	0.895	3.111				
	SI 4	0.912	3.577				
Self-efficacy	SE 1	0.856	2.212	0.870	0.870	0.911	0.719
	SE 2	0.864	2.315				
	SE 3	0.843	2.047				
	SE 4	0.829	1.910				
Personal innovativeness	PI 1	0.872	2.751	0.876	0.876	0.915	0.729
	PI 2	0.865	2.649				
	PI 3	0.827	2.024				
	PI 4	0.849	2.240				
Perceived ease of use	PEU 1	0.812	1.846	0.871	0.873	0.912	0.721
	PEU 2	0.872	2.305				
	PEU 3	0.850	2.341				
	PEU 4	0.861	2.399				
Perceived usefulness	PU 1	0.886	2.657	0.905	0.906	0.933	0.778
	PU 2	0.882	2.558				
	PU 3	0.875	2.597				
	PU 4	0.885	2.741				
Perceived risk	PR 1	0.925	2.998	0.904	0.915	0.940	0.839
	PR 2	0.931	3.299				
	PR 3	0.891	2.611				
Behavioral intention	BI 1	0.864	2.413	0.903	0.904	0.932	0.775
	BI 2	0.904	3.272				
	BI 3	0.890	3.023				
	BI 4	0.861	2.313				

VIF = variance inflation factor; α = cronbach’s alpha; CR = composite reliability; AVE = average variance extracted.

**Table 4:** Discriminant validity.

Construct	SI	SE	PI	PEU	PU	PR	BI
SI	**0.910**	0.762	0.572	0.691	0.778	0.251	0.793
SE	0.686	**0.848**	0.680	0.742	0.595	0.375	0.781
PI	0.516	0.594	**0.854**	0.705	0.566	0.317	0.704
PEU	0.625	0.647	0.618	**0.849**	0.768	0.301	0.844
PU	0.716	0.530	0.505	0.684	**0.882**	0.203	0.789
PR	−0.233	−0.335	−0.286	−0.268	−0.185	**0.916**	0.383
BI	0.727	0.692	0.626	0.750	0.716	−0.348	**0.880**

SI = social influence, SE = self-efficacy, PI = personal innovativeness, PEU = perceived ease of use, PU = perceived usefulness, PR = perceived risk, BI = behavioral intention; Main diagonal bold values are square root of average variance extracted; Fornell-Larcker criterion (below the main diagonal) and Heterotrait-Monotrait Ratio (HTMT) (above the main diagonal).

### 5.3 Evaluation of the structural model

**Figure 2:**
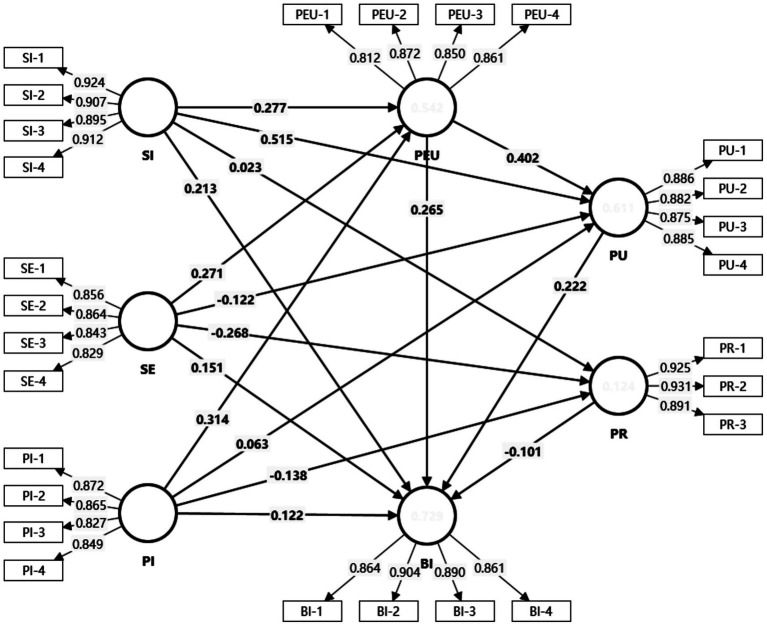
Path coefficients of the structural model.

The measurement model having been validated, the focus was on assessing the structural model. Testing of hypotheses was the main activity in this evaluation process. Table 5 has provided a descriptive overview of the hypothesis test results of the structural model. According to the analysis of the path coefficients (*β*) and significance levels (*p*-values), all the hypotheses but H1c, H2b and H3b were statistically supported, providing data support for the characteristic analysis of acceptance behavioral intentions in older adults. The visual presentation of the path coefficients of the research model is included in Figure 2. Hypothesis results:

- Social Influence: Positively affected perceived ease of use (β = 0.277, *p* < 0.001), perceived usefulness (β = 0.515, *p* < 0.001), and behavioral intention (β = 0.213, *p* < 0.001), supporting H1a, H1b, and H1d. H1c (to perceived risk) was ultimately rejected due to a non-significant p-value.
- Self-Efficacy: Positively influenced perceived ease of use (β = 0.271, *p* < 0.001) and behavioral intention (β = 0.151, *p* < 0.001), and negatively affected perceived risk (β = −0.268, *p* < 0.001), validating H2a, H2c, and H2d. However, its negative effect on PU (β = −0.122, *p* < 0.01) contradicted H2b.
- Personal Innovativeness: Positively impacted perceived ease of use (β = 0.314, *p* < 0.001) and behavioral intention (β = 0.122, *p* < 0.001), and negatively affected perceived risk (β = −0.138, *p* < 0.05), supporting H3a, H3c, and H3d. Its effect on perceived usefulness (β = 0.063, *p* > 0.05) was insignificant, rejecting H3b.
- TAM Paths: perceived ease of use positively influenced perceived usefulness (β = 0.402, *p* < 0.001) and behavioral intention (β = 0.265, *p* < 0.001), while perceived usefulness positively affected behavioral intention (β = 0.222, *p* < 0.001), confirming TAM’s validity.
- Perceived Risk: Negatively impacted behavioral intention (β = −0.101, *p* < 0.001), supporting H6.

**Table 5:** Structural model hypothesis testing using PLS algorithm and bootstrapping.

Hypothesis	Path	β	ƒ^2^	t-value	*p*-value	Decision
H1a(+)	SI ➔ PEU	0.277	0.085	6.490	***	Supported
H1b(+)	SI ➔ PU	0.515	0.321	11.972	***	Supported
H1c(−)	SI ➔ PR	0.023	0.000	0.394	0.694	Not supported
H1d(+)	SI ➔ BI	0.213	0.059	4.809	***	Supported
H2a(+)	SE ➔ PEU	0.271	0.073	5.935	***	Supported
H2b(+)	SE ➔ PU	−0.122	0.016	2.800	0.005	Not supported
H2c(−)	SE ➔ PR	−0.268	0.037	3.988	***	Supported
H2d(+)	SE ➔ BI	0.151	0.034	3.897	***	Supported
H3a(+)	PI ➔ PEU	0.314	0.134	7.262	***	Supported
H3b(+)	PI ➔ PU	0.063	0.006	1.736	0.083	Not supported
H3c(−)	PI ➔ PR	−0.138	0.014	2.575	0.010	Supported
H3d(+)	PI ➔ BI	0.122	0.030	3.528	***	Supported
H4a(+)	PEU ➔ PU	0.402	0.190	9.569	***	Supported
H4b(+)	PEU ➔ BI	0.265	0.099	6.288	***	Supported
H5(+)	PU ➔ BI	0.222	0.070	5.530	***	Supported
H6(−)	PR ➔ BI	−0.101	0.033	4.172	***	Supported

SI = social influence, SE = self-efficacy, PI = personal innovativeness, PEU = perceived ease of use, PU = perceived usefulness, PR = perceived risk, BI = behavioral intention; β = path coefficients, ƒ^2^ = effect size. ***p < 0.001.

In this study, the effects size two and the coefficient of determination R^2^ and cross-validated redundancy Q^2^ were employed to fully determine the predictive potential of the structural model (89). Tables 5, 6 contain the overall outcome of a review of these measures. In particular, PEU, PU, PR, and BI yield the R^2^ values of 0.542, 0.611, 0.124 and 0.729, respectively. Except for PR, the R^2^ values of the other three variables are all surpassed 0.5, and their corresponding Q^2^ values exceeded 0.25, suggesting they each possess moderate predictive capabilities. For the variable PR, although its R^2^ value is below 0.25, its ƒ^2^ value basically reaches 0.02, and its Q^2^ value is greater than 0, indicating that PR has weak but meaningful predictive value in the model. The structural model in this study demonstrates good predictive ability performance. Therefore, the theoretical model proposed in this study has strong predictability, emphasizing the stability and predictability of the causal relationship between variables in this research context.

**Table 6:** R^2^ and Q^2^ for all endogenous constructs.

Constructs	R-square	R-square adjusted	Q-square	Outcome
PEU	0.542	0.539	0.534	Moderate
PU	0.611	0.608	0.531	Moderate
PR	0.124	0.118	0.111	Weak
BI	0.729	0.725	0.637	Moderate

PEU = perceived ease of use, PU = perceived usefulness, PR = perceived risk, BI = behavioral intention.

### 5.4 Mediation effect test

This study employed the bootstrap method, with 5,000 resamples at a 95% confidence level, to thoroughly estimate and test the mediation effects involving perceived ease of use, perceived usefulness, and perceived risk. The detailed results are presented in Tables 7,8. First, perceived ease of use (effect size = 0.073, CI [0.043, 0.111]) and perceived usefulness (effect size = 0.114, CI [0.069, 0.163]) each played partial mediating roles between social influence and behavioral intention, while the mediating effect of perceived risk was insignificant. Second, perceived ease of use (effect size = 0.072, CI [0.042, 0.107]) and perceived risk (effect size = 0.027, CI [0.011, 0.048]) each exerted partial mediating effects between self-efficacy and behavioral intention, whereas perceived usefulness showed no mediating effect. Finally, perceived ease of use (effect size = 0.083, CI [0.052, 0.118]) and perceived risk (effect size = 0.014, CI [0.003, 0.029]) each served as partial mediators between personal innovativeness and behavioral intention, while the mediating effect of perceived usefulness was not evident. The identification of mediating variables provides empirical evidence for unpacking the psychological mechanisms and underlying cognitive logic behind older adults’ adoption of shared service platforms outside LTCI, laying a solid foundation for subsequent theoretical dialogues.

**Table 7:** Indirect effects analysis.

Paths	Effect type	Effects	BootLLCI	BootULCI	*p*-values	Mediation
SI ➔ PU	In	0.111	0.074	0.155	0.000	Partial
	Di	0.515	0.428	0.597	0.000	
	To	0.626	0.546	0.703	0.000	
SI ➔ BI	In	0.210	0.154	0.272	0.000	Partial
	Di	0.213	0.126	0.297	0.000	
	To	0.423	0.344	0.503	0.000	
SE ➔ PU	In	0.109	0.069	0.155	0.000	full mediation
	Di	−0.122	−0.205	−0.036	0.005	Not supported
	To	−0.013	−0.100	0.072	0.780	
SE ➔ BI	In	0.096	0.049	0.147	0.000	Partial
	Di	0.151	0.074	0.225	0.000	
	To	0.247	0.169	0.324	0.000	
PI ➔ PU	In	0.126	0.085	0.174	0.000	full mediation
	Di	0.063	−0.009	0.134	0.083	Not supported
	To	0.189	0.112	0.267	0.000	
PI ➔ BI	In	0.139	0.099	0.184	0.000	Partial
	Di	0.122	0.053	0.189	0.000	
	To	0.261	0.189	0.333	0.000	
PEU ➔ BI	In	0.089	0.055	0.125	0.000	Partial
	Di	0.265	0.185	0.348	0.000	
	To	0.354	0.274	0.441	0.000	

In = Indirect effects, Di = Direct effects, To = Total effects; SI = social influence, SE = self-efficacy, PI = personal innovativeness, PEU = perceived ease of use, PU = perceived usefulness, PR = perceived risk.

**Table 8:** Specific indirect effects analysis.

Paths	Effects	BootLLCI	BootULCI	P-values	Decision
SI ➔ PEU ➔ PU	0.111	0.074	0.155	0.000	Supported
SI ➔ PEU ➔ PU ➔ BI	0.025	0.014	0.037	0.000	Supported
SI ➔ PEU ➔ BI	0.073	0.044	0.111	0.000	Supported
SI ➔ PU ➔ BI	0.114	0.070	0.163	0.000	Supported
SI ➔ PR ➔ BI	−0.002	−0.015	−0.010	0.704	Not supported
SE ➔ PEU ➔ PU	0.109	0.069	0.155	0.000	Supported
SE ➔ PEU ➔ PU ➔ BI	0.024	0.013	0.038	0.000	Supported
SE ➔ PEU ➔ BI	0.072	0.042	0.108	0.000	Supported
SE ➔ PU ➔ BI	−0.027	−0.050	−0.007	0.011	Not Supported
SE ➔ PR ➔ BI	0.027	0.011	0.047	0.004	Supported
PI ➔ PEU ➔ PU	0.126	0.085	0.174	0.000	Supported
PI ➔ PEU ➔ PU ➔ BI	0.028	0.016	0.042	0.000	Supported
PI ➔ PEU ➔ BI	0.083	0.053	0.119	0.000	Supported
PI ➔ PU ➔ BI	0.014	−0.002	0.032	0.099	Not Supported
PI ➔ PR ➔ BI	0.014	0.003	0.029	0.034	Supported
PEU ➔ PU ➔ BI	0.089	0.055	0.125	0.000	Supported

SI = social influence, SE = self-efficacy, PI = personal innovativeness, PEU = perceived ease of use, PU = perceived usefulness, PR = perceived risk.

## 6 Discussion

### 6.1 Primary findings

Following the research approach of “empirical testing - cognitive perspective - theoretical dialogue,” this paper will interpret the cognitive logic of the Japanese older adult willingness to accept shared services outside of LTCI step by step based on the model testing results, and attempt to explain it from a broader theoretical perspective in order to provide theoretical reference for alleviating digital inequality among the older adult.

First, this study found that the research scenario may have a shaping effect on localized behavioral intentions of technology acceptance among older adults. Most of the hypotheses in this study hold true, indicating that in the emerging scenario of shared services outside of LTCI, older adults’ technology acceptance behavior largely conforms to theoretical expectations (as shown in Table 5). However, not all results are universally applicable across scenarios. According to previous research studies in the healthcare-related field, the behavioral intentions of older users may not be influenced by either perceived ease of use (27–29) or perceived usefulness (30, 31). Also, the positive effect of the perceived ease of use on the perceived usefulness was not consistent (28). Furthermore, the effects of variables such as social influence, self-efficacy, personal innovativeness, and perceived risk on behavioral intentions may also vary depending on the context (32–36). Existing research often limits itself to explaining the impact of these variables on technology acceptance behavioral intentions based on the behavioral characteristics of the older adult within its own research context, lacking cross-contextual discussion. This paper acknowledges the universal theoretical value of technology acceptance models, but also argues that when technology is embedded in different emerging service scenarios, the path of action of the same theoretical model may produce local differences. This also suggests that contextual characteristics may be an important reason for the partial differentiation in technology acceptance behavioral intentions among the older adult. This research argument may resonate with the currently highly regarded research literature (20, 96, 97).

Based on this conclusion, we can explore this further with examples. From the perspective of the older adult, medical care and social entertainment are both frequently encountered scenarios in their daily lives, and services in these two scenarios are of great interest to them. Even when using the same smartphone, the older adult acceptance behavioral intentions of healthcare and social entertainment technology platforms may differ. Medical care platforms inevitably involve cumbersome and complex procedures during user registration and use, and may even infringe on deep personal privacy; while social entertainment platforms offer simpler content and generally do not involve privacy issues. Influenced by the characteristics of the scenarios, when faced with medical care platforms, the older adult may be more inclined to be influenced by recommendations from those around them, or even seek assistance from others; when faced with social entertainment platforms, they are more willing to try them independently, and their subjective desire not to fall behind may be stronger. In this case, individual characteristics become the dominant factor, and seeking help from others is only an alternative when they cannot operate independently. Furthermore, even within the same healthcare service scenario, the platforms corresponding to public care and market-based care may elicit different acceptance responses from the older adult. Public care services led by the government have high penetration and long-term use, leading to stronger recognition and trust among the older adult. When using such platforms, self-efficacy and personal innovativeness may play a major role, while recommendations from others may only be supplementary. However, market-based nursing care platforms are mostly operated by emerging companies with a limited audience and low public awareness. Coupled with the older adult lack of understanding of the services offered, they are generally wary. In this context, social influence becomes a key factor; the older adult are more willing to refer to recommendations from existing users, while the role of personal traits is weakened or even negligible (even though empirical results show that individual traits such as self-efficacy and personal innovativeness have a significant positive impact on behavioral intentions, their path coefficients may be lower than the strength of social influence factors, as shown in Table 5). This study suggests that the localized differences in technology acceptance behavioral intentions among the older adult in different scenarios may essentially stem from scenario-specific differences in individual cognitive mechanisms. When the older adult perceive the service scenario behind the technology as complex, difficult to understand, and unfamiliar, they may immediately choose to follow the advice of others; while when they feel the service scenario is simple and widely accessible, they may be more willing to exhibit characteristics such as self-efficacy and personal innovativeness.

Contextual characteristics may be a significant reason for the partial differentiation in technology acceptance behavioral intentions among the older adult. This conclusion echoes contingency theory in management, which posits that “there is no single best universal explanatory model suitable for all situations.” The effectiveness and behavioral impact of any theoretical approach depend on the external environment and specific contextual characteristics of the research subjects. While this theory may seem to conflict with the universality of technology acceptance models, they are not contradictory. When individuals face various emerging technological services, regardless of changing contexts, their underlying cognitive logic for technology acceptance remains universal, rationally weighing the two core dimensions of “whether the technology is useful” and “whether it is convenient to use.” This is the core reason for the universality of technology acceptance models; this universality can be seen as “structural universality.” However, when individuals focus on technological services closely related to their lives, specific contextual characteristics may begin to reshape their localized technology acceptance behavioral intentions. We can imagine technology acceptance behavior under two different contextual characteristics as two intersecting but not completely overlapping circles. The overlapping portion illustrates the universality of the technology acceptance model, while the non-overlapping portion precisely illustrates the reshaping effect of contextual characteristics on behavioral intentions. Therefore, considering the characteristics of different scenarios plays an indispensable role in understanding the technology acceptance behavioral intentions of older adults and provides new directions for thinking about how to reduce digital inequality.

Secondly, the empirical results show that among all variables influencing behavioral intentions, perceived ease of use has the highest path coefficient, making it the most important factor determining the older adult technology acceptance behavioral intentions (as shown in Table 5). This indicates that, when faced with a shared service platform outside of LTCI, the older adult are most concerned with the platform’s simple operation and easy-to-learn cognitive logic. This extreme focus on the platform’s ease of use reflects the potential weakness in digital cognition and operational skills among the older adult in Japan. The number of older adult with technical skills who can skillfully use the platform is relatively small, while the proportion of older adult lacking the corresponding skills and unable to operate independently is much higher, forming a significant group differentiation. This digital inequality may be one of the important reasons for the current limited number of older adult users on this platform. Next, hypotheses H1d, H2d, and H3d all hold, emphasizing the promoting effects of social influence, self-efficacy, and personal innovativeness on the older adult acceptance behavioral intentions in this shared service scenario (as shown in Table 5). This is consistent with the results in other research scenarios (33, 34, 56, 60, 63, 65, 77, 82). It is worth noting that the coefficient of social influence on behavioral intention is 1.56 times the mean coefficient of self-efficacy and personal innovativeness, and its influence intensity is significantly higher than that of individual characteristics. The cognitive logic behind this behavior may be related to the specific scenario of shared services outside LTCI. These shared services are mostly dominated by startups and have low market trust; at the same time, the service process is relatively complex, and a large amount of personal information needs to be filled in during the registration process in order to achieve accurate matching of care resources. In addition, the older adult may have a sense of unfamiliarity due to insufficient understanding of the concept of services outside LTCI. The combination of multiple factors makes this scenario complex and unfamiliar, which means that when older adult come into contact with the platform, they may first think of the recommendations and usage suggestions of those around them.

Finally, this study also found that perceived ease of use, perceived usefulness, and perceived risk are three mediating variables that serve as intermediate bridges between external factors and behavioral intentions. According to the empirical test results (as shown in Table 8): (1) Social influence, self-efficacy, and personal innovativeness can all reach behavioral intentions through perceived ease of use, which further highlights the primary role of perceived ease of use. When faced with a shared service platform outside of LTCI, regardless of the factors that influence the older adult, the final achievement of their technology acceptance behavior is highly dependent on the filtering of the underlying psychological mechanism of perceived ease of use. Perceived ease of use is likely to become the “cognitive master switch” for the older adult to measure and transform all external influencing factors and technology acceptance behavioral intentions. (2) Perceived usefulness is a cognitive bridge between social influence and behavioral intentions, but cannot serve as a mediating mechanism between self-efficacy, personal innovativeness, and behavioral intentions. Because the older adult are aware that their remaining lifespan is limited, they value immediate utility and actual returns more than novelty when they come into contact with new technologies. This high attention to perceived usefulness is likely the result of recommendations from those around them, rather than determined by individual characteristics of self-efficacy and personal innovativeness. As mentioned above, in this scenario, the older adult willingness to use the platform is primarily based on the recommendations and evaluations of others to establish cognition, and external opinions may be the core source of perceived usefulness. Due to the complexity and unfamiliarity of the scenario, the older adult may not have the idea of relying on their own abilities to perceive the value of the platform from the beginning, and self-efficacy and personal innovativeness are difficult to play a role. This is also the reason why the research hypotheses H2b and H3b are not valid. Therefore, in such a scenario, the older adult may only pay attention to the platform recommendations of others, focus on understanding the functional value and practical advantages of the platform, in order to answer their own question of “why use it,” thereby promoting their willingness to accept it. (3) Perceived risk is the cognitive bridge between self-efficacy and individual innovation and behavioral intention, respectively, but cannot become the mediating mechanism between social influence and behavioral intention. The reason for this result may also be related to the scenario characteristics of shared services outside of LTCI. As mentioned above, the platform is operated by a company, and a large amount of personal information needs to be filled in during the use process, which makes the older adult have a strong sense of risk from the outset. These concerns, stemming from subjective preconceptions, are difficult to eliminate through external verbal explanations or persuasion, ultimately leading to a psychological state of “remaining wary even when recommended by others,” which is why hypothesis H1c is invalid. Therefore, in such complex and unfamiliar scenarios, older adults may ultimately exhibit their individual characteristics, demonstrating higher self-efficacy and personal innovativeness. When faced with such platform service demands, they exhibit a “It’s better to rely on oneself than to seek help from others” cognitive logic, thus forming higher self-efficacy and personal innovativeness, and generating behavioral intentions through the mediating mechanism of perceived risk.

### 6.2 Theoretical implications

Based on the above discussion, this study believes it will make the following key theoretical contributions: First, the single cognitive load of new technology in old scenarios and the dual cognitive load of new technology in new scenarios are crucial to whether older adults’ technology acceptance behavioral intentions undergoes partial reshaping. When older adults face new technologies and simple, familiar traditional scenarios, their cognitive logic is “the technology is unfamiliar, but the service content is familiar.” In this case, the universality of the technology acceptance model often plays a dominant or even decisive role in adoption behavioral intentions. However, when they face new technologies and complex, unfamiliar scenarios (including unfamiliarity due to insufficient conceptual understanding), they may experience digital anxiety brought about by the new technology and service anxiety brought about by the new scenario. Under this cognitive premise of dual anxiety, the partial universality of the technology acceptance model may be challenged, and the scenario constraints emphasized by contingency theory begin to have a partial reshaping effect on older adults’ technology acceptance behavioral intentions. It should also be emphasized that this reshaping effect is “partial” rather than “fundamental.” After all, in the scenario of this study, most research hypotheses still hold, consistent with the test results of most previous studies on older adults’ technology acceptance behavioral intentions.

Secondly, perceived ease of use acts as a “cognitive master switch” for older adults’ technology acceptance behavioral intentions. In other words, whenever technology acceptance is involved, regardless of the context, perceived ease of use exhibits strong universality among older adults. This finding emphasizes the “first principle” of perceived ease of use in the cognitive logic of older adults. This study also suggests that under the dual cognitive anxiety of new technologies and scenarios, older adults initially have a strong subjective urge to heed recommendations and evaluations from others, while their own individual characteristics are actively downplayed or even rendered ineffective. This conclusion also provides a direction for mitigating digital inequality in new service scenarios, clarifying that the priority for mitigation should first consider the key factor of social influence, rather than individual characteristics.

Finally, in this type of research scenario, the “external social construction” of perceived usefulness and the “endogenous individual defense” of perceived risk may be unique cognitive logics in the technology acceptance behavioral intentions of older adults. In such scenarios, influenced by the preconceived notion of dual anxiety, older adults may prioritize relying on recommendations and evaluations from others, rather than actively trying and making their own judgments to form a perception of the usefulness of sharing platforms, thus leading to acceptance behavioral intentions. The cognitive logic of acquiring perceived usefulness through external social influence—the “external social construction”—may be a specific behavioral intentions in this type of scenario in the technology acceptance behavioral intentions of older adults. Similarly, compared to perceived usefulness, perceived risk is difficult to dispel through verbal explanations and simple introductions. Older adults generally have a mindset that “persuasion from others is still difficult to dispel concerns,” prompting them to rely more on their own abilities when using technology. Based on this cognitive logic, even when facing dual anxiety, older adults may be more inclined to rely on “endogenous individual defenses,” using self-efficacy and personal innovativeness to weaken risk perception, ultimately forming a willingness to use the technology.

### 6.3 Management implications

The core value of theoretical research lies in guiding practice. This study will offer targeted management suggestions to operators and managers of technology platforms serving the older adult. Firstly, in platform operation and management, managers should abandon a singular technological mindset, avoid the cognitive pitfalls of “technology reductionism,” and pay attention to the scenario-based cognitive characteristics formed by older adult users based on their overall understanding of the industry. Although key factors in the technology acceptance model, including perceived ease of use, perceived usefulness, social influence, self-efficacy, personal innovativeness, and perceived risk, are universally applicable to driving older adult acceptance behavioral intentions, they cannot be blindly applied in practice without considering the company’s industry attributes and business scope. Ignoring scenario and industry differences will make it difficult to accurately grasp the underlying logic of changes in older adult user behavioral intentions in the early stages of platform launch, and may even lead to deviations in the overall operational strategy due to misjudging core driving factors. Therefore, when formulating platform promotion strategies, managers must fully consider the dual cognitive burden of the older adult regarding both the technology platform and the service model.

Secondly, when a platform represents a completely new scenario involving new technologies and services, administrators should focus on the contingent reshaping effect of the service scenario on the older adult technology acceptance behavioral intentions and adjust their strategies accordingly. In the scenario of this study, services outside of LTCI are a product of the Japanese government’s rational assessment and optimization of its LTCI policy in response to the deep aging crisis, and an effective supplement to the traditional LTCI service system. However, for older adult users, “services outside of insurance” are a completely new concept relative to the traditional “services within insurance,” and the information gap can easily lead to unfamiliarity and cognitive barriers. In fact, in terms of service content, the platform’s services overlap and are substitutable with traditional housekeeping and home care services. Based on this, during platform operation, administrators must consider both the cognitive novelty brought by new technologies and the cognitive differences caused by new service concepts, implementing dual cognitive management. Only in this way can the theoretical value of the technology acceptance model be fully, meticulously, and accurately utilized, providing scientific and effective practical guidance for the construction and optimization of an age-friendly service system, thereby helping the older adult achieve successful aging. In addition, from a dynamic evolutionary perspective, the attributes of service scenarios are not static. As long-term care services outside of insurance continue to penetrate the market, and as peer-to-peer communication and user reviews accumulate, the “novelty” of this service scenario will eventually fade over time. When this emerging scenario gradually transforms into a familiar, routine one in the minds of older adult users, the universality of the technology acceptance model will likely become fully apparent. Therefore, managers need to closely monitor the dynamic changes in older adult users’ cognitive shifts and adjust platform operation management strategies accordingly at different stages of industry development.

Furthermore, in the early stages of platform operation, managers must prioritize perceived ease of use as the “first principle of end-to-end operation,” rather than treating it as an equal factor to other platform-related factors. Especially in emerging service scenarios that are complex and unfamiliar to users, all operational, promotional, and user outreach efforts should revolve around this core principle of perceived ease of use. Taking social influence as an example, when promoting services and building positive word-of-mouth through referrals from friends and family, community outreach, and experiential activities, the overall activity plan must prioritize the integration of ease of use, rather than focusing solely on the platform’s interface. Managers should organically integrate the comprehensibility of the service scenario with the ease of use of the technical platform, ensuring that this dual perception of ease of use is accurately ingrained in the minds of older adult users. Only when seniors perceive the service scenario as easy to understand and participate in, and the platform as simple and easy to use, will they genuinely recognize the service’s value and develop a willingness to use it. Conversely, if the initial promotional phase fails to quickly answer the seniors’ questions of “What is the activity content?” and “How does it relate to them?,” an excessively high cognitive barrier will directly hinder their experience of the platform’s functions.

Finally, when promoting their platforms in new technology and service scenarios, companies may need to grasp the influence of social influence and individual characteristics (such as self-efficacy and personal innovativeness) on the psychology of older adults’ acceptance of technology, and formulate targeted strategies to enhance their willingness to use the platform. On the one hand, they can leverage social impact to create external driving forces, using communities and family and friends to build positive examples and a positive word-of-mouth atmosphere, conveying the platform’s value and guiding older adults to accept and use it. On the other hand, they can emphasize the intrinsic motivation brought by individual characteristics, improving the digital literacy of older adults through digital knowledge lectures, hands-on experiences, and other activities, stimulating their self-efficacy and individual creativity, reducing perceived risks associated with the platform, and thus encouraging users to shift from passive acceptance to active acceptance. At the same time, given the common wariness among older adults, companies also need to build a comprehensive risk control system in terms of risk communication, complaint mechanisms, and honorary qualifications to reduce their perceived risks. Therefore, a systematic platform operation strategy of “external guidance, internal stimulation, and risk control” can effectively overcome the cognitive barriers older adults face when encountering new service scenarios, ensuring the successful implementation and continuous promotion of technology applications.

### 6.4 Limitations and future research

Although this study offers a large amount of explanation regarding the factors that influence the behavioral intentions of older adults in Japan to buy shared services beyond LTCI, it has some limitations. These limitations do not only indicate the scope of the present study but also give guidelines on how future academic studies should be conducted.

To begin with, in the area of sample choice, the present study narrowed down to the Japanese older adult population. Since the process of aging differs greatly among various countries and even among various regions, our results cannot be directly applicable to the rest of the world. Thus, the scope of data gathering in future studies is to expand on the variety of specific situations to prove the relevance and usefulness of our theoretical framework to aging. Second, this research was conducted on Tokyo region of Japan due to the scarcity of sources. The limitation of this study is geographic in nature, and might affect the validity of the study outcomes due to lack of adequate sampling. To further improve the accuracy and dependability of the findings, future studies should gather information from a wider variety of regions to address this restriction. Ultimately, this study used a cross-sectional analysis approach. This method makes it challenging to expose the dynamic modifications of these aspects over the day, despite being effective in identifying the factors that influence behavior for expressed support platforms. Future studies should choose a longitudinal research method to explore the growing habits of older individuals ‘purchasing behavior.

## 7 Conclusion

In an era where technological change drives social development, digital inequality is prevalent among the older adult. How to help older adults alleviate digital inequality and achieve successful aging has become a crucial issue of common concern for academia, government, and business. This study uses shared services outside of LTCI as its research scenario, employing a technology acceptance model as its core foundation. It introduces key variables such as social influence, self-efficacy, personal innovativeness, and perceived risk to construct an expanded model of technology acceptance among older adults, and empirically examines their technology acceptance behavior patterns. Following the approach of “empirical testing - cognitive perspective - theoretical dialogue,” based on the behavior characteristics presented by the empirical results, this study delves into the underlying cognitive logic behind older users’ technology acceptance behavioral intentions, providing theoretical explanations and practical suggestions to alleviate digital inequality among the older adult.

This study will make the following theoretical and practical contributions. Theoretically, this study keenly focuses on the subtle differences behind the apparent similarities in technology acceptance behavioral intentions research among older adults, aiming to unravel the question of why older users’ technology adoption behavioral intentions exhibits localized differentiation. Through a cross-scenario comparative perspective, it explores the causes of these localized differences in older adults’ technology acceptance behavioral intentions. The study finds that these localized differences may stem from differences in their understanding of the accompanying service scenarios; that is, the characteristics of the service scenarios can have a localized reshaping effect on older adults’ technology acceptance behavioral intentions. To reasonably explain the above research conclusions, this study dialectically integrates the “scenario-based behavioral intentions” perspective emphasized by contingency theory in organizational behavioral intentions with the “universal consensus” of technology acceptance models. The study argues that these two perspectives are not contradictory but rather organically integrated within the research scenario, and, combined with empirical test results, systematically explains the inherent rationality of this integration.

From a practical perspective, this study, based on theoretical analysis, offers management insights for operators of older adult technology service platforms. In the initial stages of platform operation, managers not only need to grasp the core factors influencing the older adult technology acceptance behavioral intentions, but also, based on their own industry attributes and service boundaries, clearly understand that the essence of the older adult acceptance and use of the technology platform is the satisfaction of the supporting service needs behind the platform. The differentiated service scenario perceptions of older adult users may have a partial reshaping effect on their technology acceptance behavioral intentions. Therefore, managers need to consider both the older adult technological and service perceptions, implement dual-perception management, and dynamically adjust operational strategies according to changes in scenarios. In the initial stage of platform promotion, adhering to the “integrated marketing” concept, perceiving ease of use throughout the entire promotion process is crucial. By balancing the understandability and participation of service scenarios with the ease of operation of the technology platform, the dual cognitive anxiety of the older adult facing new technologies and new service scenarios can be alleviated, effectively increasing their willingness to accept the platform. Simultaneously, to optimize resource allocation and avoid wasting operational costs, companies should adopt precise and differentiated promotion strategies. By leveraging word-of-mouth within communities to generate external guidance, and through digital literacy training and hands-on experiences, we can activate the self-efficacy and creativity of older adult users, thereby stimulating their intrinsic motivation to use the platform. At the same time, we can build a comprehensive risk control system to alleviate the concerns of the older adult about using the platform, and break down promotion barriers with a systematic operation strategy to help the age-friendly technology platform be implemented and popularized.

## Data Availability

The raw data supporting the conclusions of this article will be made available by the authors, without undue reservation.

## Footnotes

1 Ichirou Co. (https://corp.ichirou.co.jp/) was officially established in Tokyo, Japan, in April 2017. Its direct competitor is CrowdCare Corporation, and both companies provide services through an online matching model. In March 2024, the company’s CEO, Tomoki Mizuno, co-founded the “Care-related Service Business Association” alongside 10 enterprises involved in services outside of long-term care insurance. This initiative plays a crucial leading role in promoting the deep integration of the shared economy with services outside of long-term care insurance.

2 As of March 2024, Ichirou Co. had dispatched only 43,563 online support services, with 4,957 registered partners and 3,146 registered service users. This suggests that the current number of older Japanese adults utilizing these online shared services is relatively small.

3 According to current Japanese government regulations, citizens aged 40 and above are required to enroll in long-term care insurance and begin paying premiums. This signifies that people are beginning to pay attention to long-term care insurance and related services and are developing a comprehensive understanding of them. Although the 50–59 age group has not yet crossed the internationally accepted threshold for old age, this study is a cognitive intention study prior to technology adoption, rather than a clinical care study after disability. Therefore, it is reasonable to conduct a sample survey targeting “pre-older adult” individuals who are approaching old age, have stable cognitive maturity, and possess digital literacy.

4 Criteria for eliminating invalid questionnaires: (1) If all respondents select the same option or show obvious periodic patterns in the scale, it is judged as a regular invalid response; (2) If more than 20% of the total items are missing, it is judged as an incomplete questionnaire and eliminated; (3) If the respondents are under 50 years old, they are considered as not being eligible for the survey and are eliminated.
